# Systems-Level Analysis of Genome-Wide Association Data

**DOI:** 10.1534/g3.112.004788

**Published:** 2013-01-01

**Authors:** Charles R. Farber

**Affiliations:** Center for Public Health Genomics, Departments of Medicine (Division of Cardiology) and Biochemistry and Molecular Genetics, University of Virginia, Charlottesville, Virginia 22908

**Keywords:** genome-wide association study (GWAS), systems biology, coexpression network, osteoporosis

## Abstract

Genome-wide association studies (GWAS) have emerged as the method of choice for identifying common variants affecting complex disease. In a GWAS, particular attention is placed, for obvious reasons, on single-nucleotide polymorphisms (SNPs) that exceed stringent genome-wide significance thresholds. However, it is expected that many SNPs with only nominal evidence of association (*e.g.*, *P* < 0.05) truly influence disease. Efforts to extract additional biological information from entire GWAS datasets have primarily focused on pathway-enrichment analyses. However, these methods suffer from a number of limitations and typically fail to lead to testable hypotheses. To evaluate alternative approaches, we performed a systems-level analysis of GWAS data using weighted gene coexpression network analysis. A weighted gene coexpression network was generated for 1918 genes harboring SNPs that displayed nominal evidence of association (*P* ≤ 0.05) from a GWAS of bone mineral density (BMD) using microarray data on circulating monocytes isolated from individuals with extremely low or high BMD. Thirteen distinct gene modules were identified, each comprising coexpressed and highly interconnected GWAS genes. Through the characterization of module content and topology, we illustrate how network analysis can be used to discover disease-associated subnetworks and characterize novel interactions for genes with a known role in the regulation of BMD. In addition, we provide evidence that network metrics can be used as a prioritizing tool when selecting genes and SNPs for replication studies. Our results highlight the advantages of using systems-level strategies to add value to and inform GWAS.

Genome-wide association studies (GWAS) have revolutionized complex disease genetics. In just the last few years, GWAS have been used to identify hundreds of variants affecting a diverse range of common diseases and disease associated quantitative traits (for a summary, see http://www.genome.gov/gwastudies/). Although GWAS have proven extremely effective at identifying common variants with relatively large effects, the first wave of data suggests that for many diseases, this class of variation accounts for only a small fraction of the genetic risk. For example, a large-scale, meta-analysis of ∼32,000 individuals identified 56 loci associated with bone mineral density (BMD), a strong predictor of osteoporotic fracture. However, in aggregate these single-nucleotide polymorphisms (SNPs) only explained 5.8% of the variance in femoral neck BMD (Estrada *et al.* 2012).

It is possible that for most diseases, the missing heritability is attributable to a combination of many more common variants with increasingly smaller effect sizes and rare variants, both of which are difficult to detect with GWAS in its current form (Altshuler *et al.* 2008). It has been suggested that additional genes and biological mechanisms underlying a disease process could be extracted from GWAS data by searching lists of genes harboring nominally significant (*e.g.*, *P* < 0.05) associations. Most of the initial attempts to identify such pathways have used gene ontology (GO) and pathway-enrichment tools to compare the number of genes in a specific pathway harboring nominally significant SNPs to the number expected at random. This approach has been applied to several GWAS datasets with varying results (Askland *et al.* 2009; Baranzini *et al.* 2009; Elbers *et al.* 2009a; O’Dushlaine *et al.* 2009; Peng *et al.* 2010; Ritchie 2009; Torkamani and Schork 2009; Torkamani *et al.* 2008; Wang *et al.* 2007).

Several issues complicate pathway analysis. First, enrichment results can vary widely across software tools (Elbers *et al.* 2009b). Second, enrichment analyses are biased toward what we already know concerning pathway membership, and most predefined gene categories are very general in nature, making it more difficult to develop testable hypotheses with the goal of investigating specific disease mechanisms. Third, these strategies fail to provide information on the relationships between associated genes. Such information is critical to understanding how networks of polymorphic genes work together to promote or provide protection against disease. Recently, Baranzini *et al.* 2009 used protein−protein interaction data to address this latter point by identifying interacting partners that were nominally associated with multiple sclerosis. However, missing from this approach was the ability to incorporate network concepts with clinical information. The specific goal of this study was to address these issues.

Weighted gene coexpression network analysis (WGCNA) is a widely used analytical method that identifies functional connections between genes using microarray gene expression data (Chen *et al.* 2008; Gargalovic *et al.* 2006; Ghazalpour *et al.* 2006; Horvath *et al.* 2006; Oldham *et al.* 2008; van Nas *et al.* 2009; Winden *et al.* 2009). WGCNA groups genes into modules on the basis of their coexpression similarities across a population of samples. The resulting modules have been shown to be comprised of genes that share similar functions or are involved in the same pathway [as examples: (Ghazalpour *et al.* 2006; Horvath *et al.* 2006; Oldham *et al.* 2008; van Nas *et al.* 2009)]. The advantage of WGCNA is that connections between genes can be established in an unbiased manner using disease-relevant expression data.

In the present work we used WGCNA to perform a systems-level analysis of GWAS data. The analysis was performed by combining SNP-level association data from a large BMD GWAS with microarray expression data from a disease-relevant cell type from subjects with known BMD status (low *vs.* high). Using WGCNA, we identified modules composed of genes that were highly interconnected with one another and displayed nominal evidence of association with BMD. Through the characterization of module content and topology, our approach identified biological mechanisms, modules, individual genes, and network concepts that likely play an important role in the regulation of BMD.

## Materials and Methods

### Converting SNP lists to gene lists using ProxyGeneLD

Several caveats complicate the conversion of a list of SNPs with association *P*-values to the assignment of gene-wide *P*-values using raw GWAS data. The primary confounders are linkage disequilibrium (LD) and biases due to gene size and the number of SNPs typed per gene. LD makes gene identification difficult because many nominally significant SNPs will be in LD with multiple genes. In addition, larger genes and genes with a greater density of SNPs typed have an increased probability of harboring nominally significant SNPs just by chance. Recently, Hong *et al.* 2009 developed an algorithm (referred to as ProxyGeneLD) that reduces biases by accounting for LD when annotating genes. ProxyGeneLD works by identifying clusters of GWAS SNPs (referred to as proxy clusters) in high LD (r2 ≤ 0.80) using HapMap data. It then assigns proxy clusters and singleton SNPs (that did not group within a proxy cluster) to the nearest gene. Unadjusted gene-wide *P*-values are then calculated as the minimum of any SNP, either as a singleton or member of a proxy cluster per gene. *P*-value adjustments are made by multiplying the unadjusted *P*-value by the number of SNPs assigned to that gene.

We used precomputed *P*-values from a recently published GWAS performed by deCODE (Styrkarsdottir *et al.* 2008). These data are available for download from http://content.nejm.org/cgi/content/full/NEJMoa0801197/DC1 as individual text files. The GWAS consisted of 5,861 Icelandic subjects phenotyped for hip (HBMD) and spine (SBMD) BMD and genotyped at 301,019 SNPs (Styrkarsdottir *et al.* 2008). All SNPs for both traits were annotated using ProxyGeneLD. LD patterns were determined using CEU HapMap samples and genes were defined as the transcript plus a 1-kbp extension upstream to include promoter regions. *P*-values were assigned to a total of 16,878 genes. Genes with an adjusted *P* ≤ 0.05 for at least one of the two BMD traits were referred to as the nominally significant GWAS geneset (NSGG).

### GO and pathway-enrichment analysis

We performed GO and pathway-enrichment analysis for the NSGG and network modules by using the Database for Annotation, Visualization and Integrated Discovery [DAVID (Dennis *et al.* 2003; Huang da *et al.* 2009)]. Each analysis was performed using the functional annotation charting and functional annotation clustering options. Functional annotation charting tests each individual GO or pathway term for enrichment. In contrast, functional annotation clustering combines single categories with a significant overlap in gene content and then assigns an enrichment score (ES; defined as the –log10 of the geometric mean of the *P*-values for each single term in the cluster) to each cluster, making interpretation of the results more straightforward. Functional annotation clustering cannot be performed for more than 3000 genes. Because the NSGG contained 3083 genes, we used to top 3000 ranked on adjusted *P*-value for the analysis. The search was limited to KEGG and Biocarta pathways, PFAM protein domains, and GO terms in the “Molecular Function,” “Biological Process,” and “Cellular Component” categories. Single categories were considered significantly enriched at a false discovery rate (FDR) ≤ 5%. To assess the significance of functional clusters, we created 10 sets of 3000 genes randomly selected from the aforementioned list of 16,878 genes with assigned *P*-values. Functional annotation clustering was performed for all 10 random gene sets. The max random ES was 2.75. Therefore, we used an ES cutoff of ≥3.0 as the threshold for significance in all analyses.

### Gene expression data processing

To generate gene coexpression networks we used previous published microarray data from 26 healthy Chinese females ages 20−45 yr, with a mean age of 27.3 yr (Lei *et al.* 2009). In this study expression profiles were generated from circulating monocytes that were isolated and purified from subjects with low (n = 12) and high (n = 14) BMD. We downloaded the Affymetrix CEL files from National Center for Biotechnology Information (NCBI)’s Gene Expression Omnibus (GSE7158; http://www.ncbi.nlm.nih.gov/geo/query/acc.cgi?acc=GSE7158). The raw data were imported and processed using the affy package (Gautier *et al.* 2004) for the R Language and Environment for Statistical Computing (Ihaka and Gentleman 1996). Robust multiarray algorithm was used to normalize and generate probe level expression data (Irizarry *et al.* 2003).

### WGCNA

Network analysis was performed using the WGCNA R package (Langfelder and Horvath 2008). An extensive overview of WGCNA, including numerous tutorials, can be found at http://www.genetics.ucla.edu/labs/horvath/CoexpressionNetwork/. To begin, we identified all probes assaying the expression of NSGG genes. To eliminate noise due to genes that were not expressed, we selected NSGG probes whose levels exceeded the median level of expression across the entire array. As part of our quality control, we performed a clustering and principal components analysis based on the expression of these probes. Two samples from the high BMD group, GSM172405 and GSM172418, were significant outliers and were removed from the analysis. A preliminary calculation of network connectivity was used to identify the most connected probe for each gene. A WGCNA network for the selected probes was generated exactly as described in (Farber 2010). GeneSignificance (GS) for the each network gene was defined as the absolute value of its Pearson correlation with BMD status. Module Membership (MM) was calculated as the Pearson correlation between each gene’s expression and its module eigengene, calculated using Singular Value Decomposition (Alter *et al.* 2000). Network depictions were constructed using Cytoscape (Shannon *et al.* 2003).

### *In silico* replication

To compare replication success rates in hubs and genes with the highest GWAS *P*-values, we used data from a second GWAS, the Framingham Osteoporosis Study [FOS (Kiel *et al.* 2007)]. The FOS GWAS consisted of 1141 subjects genotyped at ∼100,000 SNPs. We downloaded the association data [in the form of SNPs and precomputed *P*-values generated using generalized estimating equation models (Kiel *et al.* 2007)] for three BMD traits (femoral neck, lumbar spine, and trochanter) from the database of Genotype and Phenotype at NCBI (http://www.ncbi.nlm.nih.gov/sites/entrez?Db=gap). SNP lists for each of the three traits were converted to gene lists using ProxyGeneLD precisely as described previously. A gene was considered successfully replicated if it had an unadjusted *P* ≤ 0.05 for at least one of the three BMD traits. The percentage of successfully replicated genes was calculated in the blue, magenta, greenyellow, and brown modules for the top 20%, 10%, and 5% of genes based on intramodular connectivity (k.in). These rates were compared with those for the top 20%, 10%, and 5% of GWAS network genes selected based on adjusted *P*-value from the deCODE (Styrkarsdottir *et al.* 2008) GWAS or GS.

## Results

### Identifying genes with nominally significant genome-wide associations

**Figure 1:**
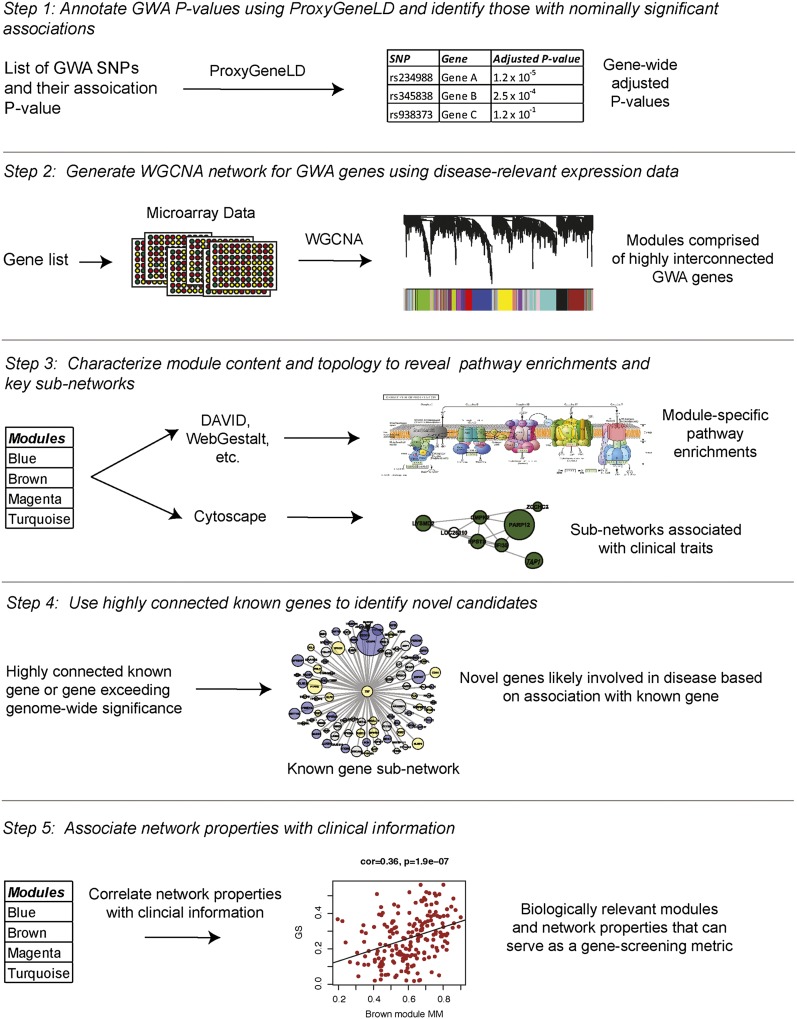
Overview of the systems-level analysis of GWAS data.

An overview of the systems-level analysis of GWAS data are presented in Figure 1. The first step in the analysis was the identification of genes displaying evidence of association using data from a BMD GWAS [n = 5861 (Styrkarsdottir *et al.* 2008)]. We used the ProxyGeneLD algorithm (Hong *et al.* 2009), which takes LD patterns into account when assigning SNPs to genes and adjusts for gene length and SNP density biases (see *Materials and Methods*), to generate gene-wide adjusted *P*-values for two osteoporosis-related traits, HBMD and SBMD. Gene-wide *P*-values were calculated for a total of 16,878 genes. Of these, 1777 and 1861 had gene-wide adjusted *P* ≤ 0.05 for HBMD and SBMD, respectively. By combining the two lists, 3083 unique genes were identified with adjusted *P* ≤ 0.05 for at least one of the BMD traits. We refer to these genes as NSGG.

To determine whether gene length and SNP density were potential confounders in the NSGG, we calculated the correlation between these two variables and HBMD unadjusted (defined as the minimum *P*-value for proxy clusters and single SNPs assigned to a particular gene) and adjusted *P*-values. As described previously, 1777 genes had adjusted *P* ≤ 0.05. In contrast, 5228 genes had unadjusted *P* ≤ 0.05. In the latter gene set, we observed a strong correlation between unadjusted *P* and gene length (r = 0.46, *P* = 0) and SNP density (r = 0.50, *P* = 0). However, this correlation was not observed after adjustment for gene length (r=-0.01, *P* = 0.88) or SNP density (r = −0.01, *P* = 0.74). Thus, our network analysis of GWAS genes should not be influenced by these systematic biases.

### Conventional pathway enrichment fails to pinpoint specific biological mechanisms

We next determined whether the NSGG was enriched for “biological themes” using the conventional approach of GO and pathway enrichment analysis. DAVID (Dennis *et al.* 2003; Huang da *et al.* 2009) was used for this analysis, although we also used WebGestalt (Zhang *et al.* 2005) and observed similar results. A total of 24 individual terms, all of which were GO categories, were significantly enriched in the NSGG at an FDR ≤ 5% (Supporting Information, File S1). The most significant term was protein binding (GO:0005515; FDR = 1.7 × 10^−10^). Other significant categories included developmental process (GO:0032502; FDR = 9.5 × 10^−5^), cation binding (GO:0043169; FDR = 2.5 × 10^−3^), and cell differentiation (GO:0030154; FDR = 2.7 × 10^−2^).

DAVID also generates category clusters by condensing sets of related terms (Dennis *et al.* 2003; Huang da *et al.* 2009). This condenses redundant categories, identifies terms containing a smaller number of genes that on their own would require higher fold enrichments to reach statistical significance, and makes interpreting the results much easier. Each cluster receives an ES, which is defined as the geometric mean (on a –log10 scale) of the *P*-values for all single terms in the cluster. A total of 32 clusters had ESs > 1.3 (equivalent to a nominal *P* ≤ 0.05); however, it was unclear whether this was an appropriate significance cutoff. To determine the distribution of ESs observed using a set of random genes we created 10 sets of 3000 genes randomly selected from the whole genome and ran each through DAVID. ESs for the random gene sets ranged from 1.36 to 2.75. Therefore, we selected an ES cutoff of ≥3.0. Using this threshold, a total of five significant clusters were identified in the NSGG (Table 1 and File S2). The top GO terms in each of the five clusters were “intracellular part,” “metal ion binding,” “developmental process,” “intracellular organelle part,” and “organelle inner membrane.” These data indicate that the NSGG is enriched for groups of genes sharing similar functionality; however, because the identified categories are very general in nature this analysis does little to pinpoint specific biological mechanisms underlying variation in BMD.

**Table 1:** Gene category and pathway enrichment analysis of NSGG genes

Functional Group	Top GO Term	Top Term FDR	ES*^a^*
1	GO:0044424∼intracellular part	9.5 × 10^−7^	6.3
2	GO:0046872∼metal ion binding	1.3 × 10^−4^	5.9
3	GO:0032502∼developmental process	9.5 × 10^−5^	5.6
4	GO:0044446∼intracellular organelle part	5.5 × 10^−2^	4.2
5	GO:0019866∼organelle inner membrane	2.1 × 10^−1^	3.3

a ES, enrichment score defined as the –log10 (geometric uncorrected *P*-value for all single categories) for each DAVID cluster.

### Generation of a weighted gene coexpression network for NSGG genes

**Figure 2:**
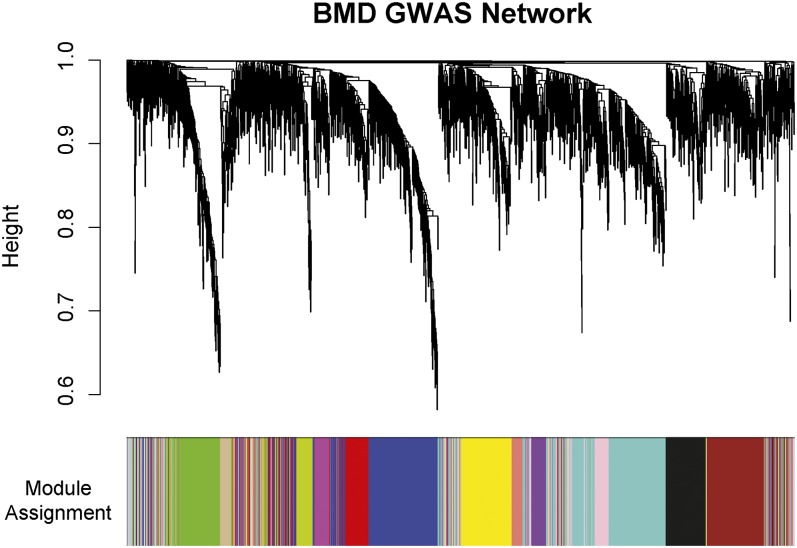
WGCNA coexpression network composed of BMD GWAS genes. Shown is the hierarchical clustering dendogram for all 1918 genes used in the analysis. Each line is an individual gene. Genes were clustered based on a dissimilarity measure (1 − TOM). The branches correspond to modules of highly interconnected groups of genes. The tips of the branches represent genes that are the least dissimilar and thus share the most similar network connections. Below the dendogram each gene is color coded to indicate its module assignment.

WGCNA reveals connections between genes using microarray expression data by grouping genes based on a topological overlap measure [TOM (Dong and Horvath 2007; Zhang and Horvath 2005)]. Two genes have a high TOM if they are highly interconnected with the same set of genes (Dong and Horvath 2007; Zhang and Horvath 2005). To evaluate the coexpression relationships between NSGG genes in a disease-relevant context we used microarray expression profiles of purified circulating monocytes isolated from individuals with discordant levels of BMD (Lei *et al.* 2009). The dataset included 24 profiles from young (mean age = 27.3 years) Chinese females, 12 with low BMD (mean Z-score=-1.72) and 12 with high BMD (mean Z-score = 1.57). We choose to use this dataset because it represents the largest study performed to date with both expression profiles for a cell-type relevant to BMD [monocytes are precursors to bone-resorbing osteoclasts (Fujikawa *et al.* 1996)] and clinical information on the subjects. After excluding non- and lowly expressed genes we identified probes representing 1918 (62%) of the 3083 NSGG genes and applied the WGCNA algorithm to generate a GWAS network. The resulting network was composed of 13 distinct gene modules (Figure 2). Sixty-three of the genes failed to fit within a distinct group and were assigned to the “grey” module. The modules ranged in size from 40 (salmon module) to 356 genes (turquoise module). A complete list of module assignments and network metrics for all genes is included in File S3.

The WGCNA approach has been used to generate robust networks in several diverse applications (Chen *et al.* 2008; Gargalovic *et al.* 2006; Ghazalpour *et al.* 2006; Horvath *et al.* 2006; Oldham *et al.* 2008; van Nas *et al.* 2009; Winden *et al.* 2009), including experiments with a similar or smaller number of samples relative to this study (Gargalovic *et al.* 2006; Gong *et al.* 2007). Most WGCNA analyses, however, use a series of preliminary filtering steps to select the most biologically meaningful genes for network construction (Ghazalpour *et al.* 2006). In such studies, the expression data exclusively determines which genes are used in the analysis. Because our network genes were not selected entirely based on expression profiles, we wanted to ensure that the resulting modules were cohesive and robust. To test cohesiveness, we calculated the mean MM for each module. MM is the correlation between each gene in a module and its module eigengene. Thus, it is a measure of how tightly a particular gene fits into its module. The greater the mean MM for a module, the more similar the coexpression relationships are across the module. The mean MM ± SEM ranged from 0.60 ± 0.01 (brown module) to 0.74 ± 0.01 (tan module), indicating that modules consisted of genes sharing highly similar expression patterns. We addressed robustness, as described previously (Ghazalpour *et al.* 2006), by randomly splitting the dataset in half 1000 times and calculating k.in in each half. The analysis was performed for the largest (turquoise) and smallest (salmon) modules. The mean correlation ± SEM between the real and random k.in values was 0.65 ± 0.05 and 0.52 ± 0.03 in the turquoise and salmon modules, respectively. Thus, the GWAS network modules are cohesive and robust to exclusion of half the data.

### Characterization of module content reveals a key role for oxidative phosphorylation in the regulation of BMD

One way in which network analysis can inform GWAS is to expose pathway enrichments that were not observed in a large set of nominally significant genes, such as the NSGG. We expected that by parsing genes based on coexpression similarities, more refined functions would be condensed within modules, revealing enrichments for more specific processes. This would improve the process of converting a detectable enrichment into a testable hypothesis.

To determine whether specific modules were enriched for novel gene categories or pathways we repeated the DAVID analysis for each module. Of the 13 modules, five had at least one cluster with an ES ≥ 3.0. Interestingly, the turquoise module stood out as displaying detailed enrichments that were not observed in the analysis of the entire NSGG (Table 2 and File S4). In the turquoise module, significant enrichments were observed for six clusters with the following top terms “cytoplasmic part” (ES = 8.1), “mitochondrion” (ES = 7.3), “electron carrier activity” (ES = 4.9), “electron carrier activity” (ES = 4.2), “hydro-lyase activity” (ES = 3.9), and “RNA splicing” (ES = 3.5). Within each cluster there were a number of terms that were not significant in the entire NSGG, suggesting that partitioning genes into coexpression can reveal hidden enrichments.

**Table 2:** Network modules with significant DAVID enrichments

Module	Number of Genes	Top Term for Each Cluster	Top Term FDR	ES*^a^*
Pink	112	GO:0044446∼intracellular organelle part	0.78	3.1
		GO:0019538∼protein metabolic process	6.0 × 10^−2^	3.0
Black	134	GO:0043231∼intracellular membrane-bound organelle	2.0 × 10^−2^	3.0
Red	134	GO:0044429∼mitochondrial part	2.0 × 10^−2^	3.4
		GO:0044446∼intracellular organelle part	1.3 × 10^−1^	3.1
Blue	297	GO:0043231∼intracellular membrane-bound organelle	2.7 × 10^−6^	5.9
		hsa00040:Pentose and glucuronate interconversions	2.6 × 10^−6^	4.1
		GO:0005634∼nucleus	5.9 × 10^−6^	3.7
Turquoise	356	GO:0044444∼cytoplasmic part	7.1 × 10^−10^	8.1
		GO:0005739∼mitochondrion	6.0 × 10^−8^	7.3
		GO:0009055∼electron carrier activity	7.5 × 10^−5^	4.9
		GO:0009055∼electron carrier activity	7.5 × 10^−5^	4.2
		GO:0016836∼hydrolyase activity	2.0 × 10^−2^	3.9
		GO:0008380∼RNA splicing	9.2 × 10^−2^	3.5

a ES, enrichment score defined as the –log10 (geometric uncorrected *P*-value for all single categories) for each DAVID cluster.

To investigate the enrichments in more detail, we focused on a single enriched term in cluster 2, the KEGG pathway “oxidative phosphorylation” (oxphos), because it represented one of the most specific enriched terms. This single term was not enriched in the NSGG (FDR = 99.8); however, its enrichment in the turquoise module was significant (FDR = 1.1 × 10^−3^). Of the 356 turquoise module genes, 16 (4.5%) were involved in oxphos (Table 3). To determine whether this enrichment was specific to the GWAS network, we generated 100 random networks. Each network was created by selecting 3083 genes at random using the same gene filtering steps and network parameters used to construct the real network. A total of 114 of the 20,080 genes (0.6%) with unique gene identifiers on the array belonged to the KEGG oxphos pathway. As shown above 16 of the 356 turquoise (4.5%) module genes were involved in oxphos. Using a Fisher’s Exact test this enrichment is highly significant (4.5% *vs.* 0.6%; *P* = 1.8 × 10^−9^). We then performed this same test for each of 1709 modules belonging to the 100 random networks. None of the random module enrichment *P*-values exceeded the *P*-value for the real turquoise module, indicating that this enrichment is specific to the BMD GWAS network.

**Table 3:** Members of the turquoise module involved in oxidative phosphorylation

Gene	Unadjusted GWAS *P*-value	k.in*^a^* rank	k.total*^b^* rank	r*^c^*
*NDUFB6*	1.0 × 10^−2^	1	8	−0.10
*COX5B*	4.9 × 10^−3^	2	9	−0.22
*COX8A*	5.0 × 10^−3^	3	5	−0.22
*COX7A2*	4.2 × 10^−3^	6	22	−0.21
*NDUFA13*	7.8 × 10^−3^	9	27	−0.16
*ATP5J2*	9.0 × 10^−4^	14	54	−0.20
*NDUFS7*	3.2 × 10^−2^	15	60	−0.35
*COX6B1*	1.4 × 10^−2^	20	49	−0.25
*ATP5G2*	1.2 × 10^−3^	24	41	−0.13
*NDUFB1*	6.0 × 10^−3^	29	70	−0.08
*NDUFA2*	3.8 × 10^−2^	32	128	−0.39
*NDUFA11*	6.1 × 10^−3^	36	113	−0.14
*COX17*	1.0 × 10^−2^	54	199	−0.19
*NDUFV2*	8.0 × 10^−4^	55	111	−0.12
*NDUFA7*	8.0 × 10^−3^	69	252	−0.30
*ATP6V1H*	5.6 × 10^−4^	181	491	0.48

a k.in = Intramodule (the turquoise module) connectivity.

b k.total = Total network connectivity.

c r = Pearson correlation between expression of gene in monocytes and BMD status (low *vs.* high).

Oxphos genes were also among the most connected in both the turquoise module and the whole network (Table 3). In fact, the three most connected turquoise hubs were oxphos genes. In addition, of the 16 total genes, 15 were in the top 20% of genes when ranked on k.in (Table 3). Another observation was that the expression of all 15 highly connected oxphos genes was negatively correlated with BMD status (Table 3). Thus, by exploring the content of the turquoise module, we have identified an association between genetic variation in oxphos genes and BMD, determined that oxphos genes are module and network hubs, and determined that oxphos gene expression in monocytes was inversely correlated with BMD levels.

### Discovery of a turquoise submodule highly correlated with BMD status

**Figure 3:**
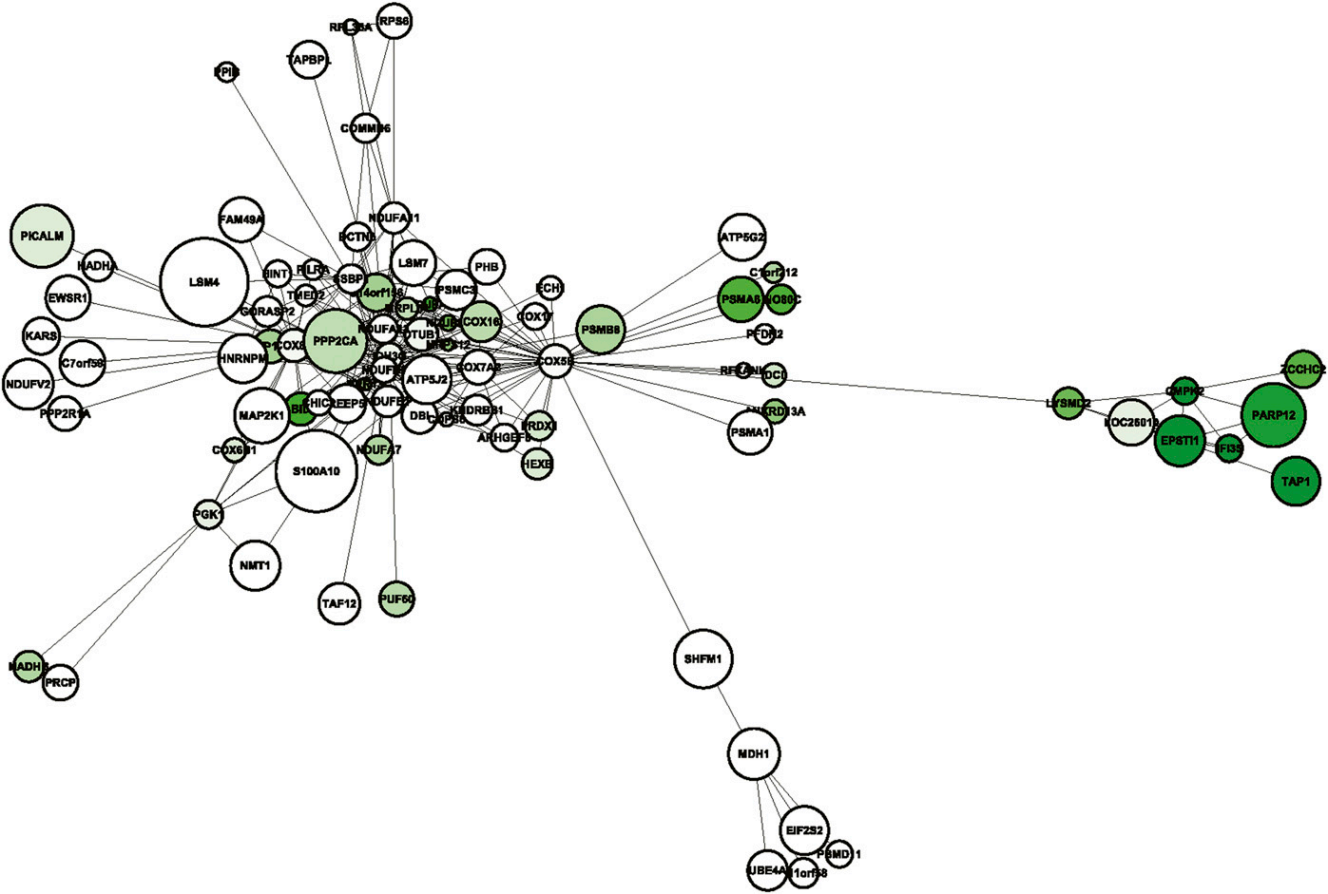
Network view of the turquoise module reveals a submodule of genes negatively correlated with BMD status. This network contains all turquoise module edges with TOM ≥ 0.15 and their corresponding nodes. Genes are shaded based on their correlation with BMD from white (no correlation) to dark green (strong negative correlation). Node sizes are proportional to each gene’s –log10 GWAS P (most significant unadjusted GWAS *P*-value for either HBMD or SBMD). The submodule of interest is on the right-hand side of the figure. Notice that this group of gene is highly interconnected and negatively correlated with BMD status.

In addition to content, module topology (the unique distribution of edges among nodes) can also be evaluated in WGCNA networks. We investigated turquoise module topology by generating a network view showing all edges with a TOM ≥ 0.15 and their corresponding nodes (Figure 3). The network consisted of 88 nodes and 256 edges. An initial inspection indicated that most nodes were grouped into a central core (containing many of the oxphos genes identified previously in this article) with two small submodules radiating from *COX5B*, an oxphos gene and the second most connected node in the module. We then overlaid information regarding the correlation between each gene’s expression and BMD status in the monocyte expression study. We suggest that correlation is a meaningful measure of biological significance, especially when considering GWAS genes, because it is likely that the correlations reflect subtle genetically-regulated differences in expression that are associated with alterations in BMD. As shown in Figure 3 most of the genes were either not correlated (nodes shaded white) or slightly negatively correlated with BMD (nodes shaded light green). None of the genes were significantly positively correlated (max correlation in the turquoise module is 0.10). Interestingly, the genes in one of the submodules were among the most negatively correlated (shaded dark green) in the turquoise module and the entire network (Table 4). One of the submodule genes, *IFI35*, was the second most negatively correlated (r = −0.58, *P* = 2.7 × 10^−3^) with BMD in the NSGG network and 4 of the 8 genes in the sub-module were in the top 50. The average correlation for this group was -0.42. To determine the probability of randomly observing a group of 8 genes this negatively correlated (Table 4) we created 10^6^ sets of 8 genes selected at random from the turquoise module. Of the random gene sets none had an average correlation more extreme than this turquoise sub-module (most negative r = −0.36).

**Table 4:** Genes comprising the turquoise sub-module

Gene	Description	Unadjusted GWAS *P*-Value	r*^a^*	r *P*-Value	Meta-analysis Distance, Kbp*^b^*	Meta-analysis *P*-Value*^c^*
*IFI35*	Interferon-induced protein 35	1.0 × 10^−2^	−0.58	2.7 × 10^−3^	742	5.1 × 10^−7^
*TAP1*	Transporter 1, ATP-binding cassette, subfamily B (MDR/TAP)	9.9 × 10^−4^	−0.48	1.7 × 10^−2^		
*EPSTI1*	Epithelial stromal interaction 1 (breast)	8.0 × 10^−4^	−0.48	1.8 × 10^−2^	510	9.8 × 10^−8^
*CMPK2*	Cytidine monophosphate (UMP-CMP) kinase 2, mitochondrial	9.6 × 10^−3^	−0.47	2.2 × 10^−2^		
*PARP12*	Poly (ADP-ribose) polymerase family, member 12	1.9 × 10^−4^	−0.42	4.0 × 10^−2^		
*ZCCHC2*	Zinc finger, CCHC domain containing 2	3.3 × 10^−3^	−0.37	7.5 × 10^−2^	172	4.9 × 10^−9^
*LYSMD2*	LysM, putative peptidoglycan-binding, domain containing 2	6.0 × 10^−3^	−0.35	9.0 × 10^−2^	564	1.4 × 10^−6^
*LOC26010*	Spermatogenesis associated, serine-rich 2-like	1.3 × 10^−3^	−0.24	2.6 × 10^−1^		

a r, Pearson correlation between expression of gene in monocytes and BMD status (low *vs.* high).

b The distance between the TSS for each respective gene and the location of a genome-wide suggestive or significant BMD association identified by (Estrada *et al.* 2012).

c The *P*-value for the associations identified by (Estrada *et al.* 2012).

Using gene information and literature searches, we found no obvious functional connection between the genes that comprised this subnetwork. However, using expression data from a panel of mouse tissues [http://www.biogps.org (Lattin *et al.* 2008; Su *et al.* 2002, 2004)] we did observe that six of the genes are expressed in osteoclasts (*EPSTl1*, *IFI35*, *PARP12*, *CMPK2*, *ZCCHC2*, and *TAP1*) and the other two are expressed in osteoblasts (*LOC26010* and *LYSMD2*). The group of osteoclast genes is also the most negatively correlated with BMD (Table 4). Next, we determined whether any of the eight genes were located in close proximity to suggestive or significant GWAS loci (*P* < 1.0 × 10^−5^) identified in a recent meta-analysis of BMD (Estrada *et al.* 2012). Interestingly of the eight, the transcription start site for four (*EPSTl1*, *IFI35*, *ZCCH2*, and *LYSMD2*) are less than 750 Kbp away from a GWAS association (Table 4). Therefore, these genes represent a highly interconnected sub-module whose expression is negatively correlated with BMD. These data together suggest they play a role in the regulation of BMD. Again, as demonstrated above, the functional interconnections between genes in this sub-module, and its correlation with BMD, was only revealed by network analysis.

### Identifying functional connections between known and novel genes

One of the advantages of our approach is the ability to identify connections between novel genes with evidence of association and those with a previously established role in disease. This information can be used in two ways. First, it can identify new pathways that a known gene may participate in and second, it can identify novel genes through “guilty by association.” To investigate the network connections for a known gene we focused on tumor necrosis factor (TNF), the most highly connected gene in the NSGG network with a known role in BMD. *TNF* was the 13th most connected gene in the entire network with a total network connectivity (k.total) of 29.0 (max k.total = 35.2). It was the 6th most connected gene in the blue module with a k.in = 27.6 (max blue module k.in = 30.8). *TNF* is known to play a prominent role in osteoclastogenesis (Lam *et al.* 2000) and several studies have found associations between *TNF* polymorphisms and BMD (Fontova *et al.* 2002; Kim *et al.* 2009). In the deCODE GWAS it was associated with HBMD and SBMD with unadjusted *P*-values of 1.2 × 10^−3^ and 1.6 × 10^−2^, respectively. The fact that *TNF* is one of hubs of a monocyte network provides additional support for the biological relevance of the GWAS network.

**Figure 4:**
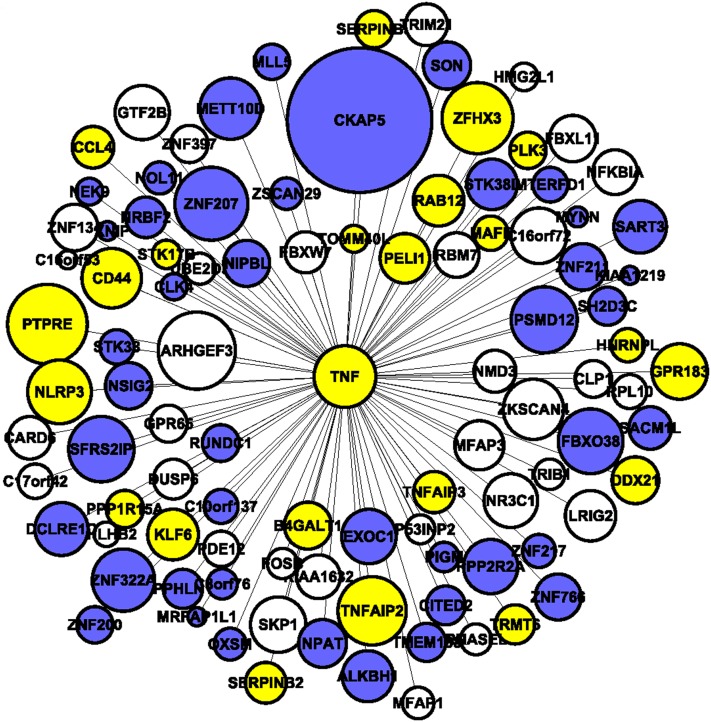
Characterizing the coexpression relationships for a highly connected known BMD gene. This TNF centered network provides a view of all edges and their corresponding nodes connected to TNF with a TOM ≥ 0.15. Genes are color coded based on their correlation with BMD; white (−0.20 < r<0.20), blue (r ≥ 0.20), and yellow (r≤-0.20). Node sizes are proportional to each gene’s –log10 GWAS P (most significant unadjusted GWAS *P*-value for either HBMD or SBMD).

We created a *TNF* submodule by identifying all edges within the blue module involving *TNF* with a TOM ≥ 0.15. The submodule contained 99 genes (Figure 4). Using DAVID we identified three significant clusters that were enriched in the sub-module with terms related to “nuclear proteins” (ES = 4.0), “gene expression” (ES = 3.6), and “regulation of transcription” (ES = 3.0) (File S5). Of the 99 genes, 47 belonged to the GO cellular component category “nucleus” (FDR = 3.82 × 10^−6^, 1.9 fold enrichment), and 32 were in the GO molecular function category “transcription factor activity” (FDR = 1.8 × 10^−4^, 3.5 fold enrichment). In support of its disease relevance the submodule included several genes with known roles in bone metabolism, such as nuclear receptor subfamily 3, group C, member 1 (glucocorticoid receptor; *NR3C1*); protein tyrosine phosphatase, receptor type, E (PTPRE); *CD44* molecule (Indian blood group; *CD44*); NLR family, pyrin domain containing 3 (*NLRP3*); FBJ murine osteosarcoma viral oncogene homolog B (*FOSB*); and dual-specificity phosphatase 6 (*DUSP6*). Thus, our network analysis rediscovered *TNF* as key intracellular signaling “hub” gene important in bone metabolism. More importantly, this network can be mined in future studies to identify novel genes that interact with *TNF* in some way (*e.g.*, are downstream targets of *TNF* signaling, etc.) to affect bone mass.

### Relating network concepts to measures of biological relevance

Exploring GWAS genes in the context of an expression network also allows one to relate network concepts, such as MM, to a measure of biological relevance. If a network property, inherent to a specific module, is associated with disease this suggests that the module serves an important biological role. It may also be possible to use the property as a gene screening tool to select genes for downstream studies.

**Figure 5:**
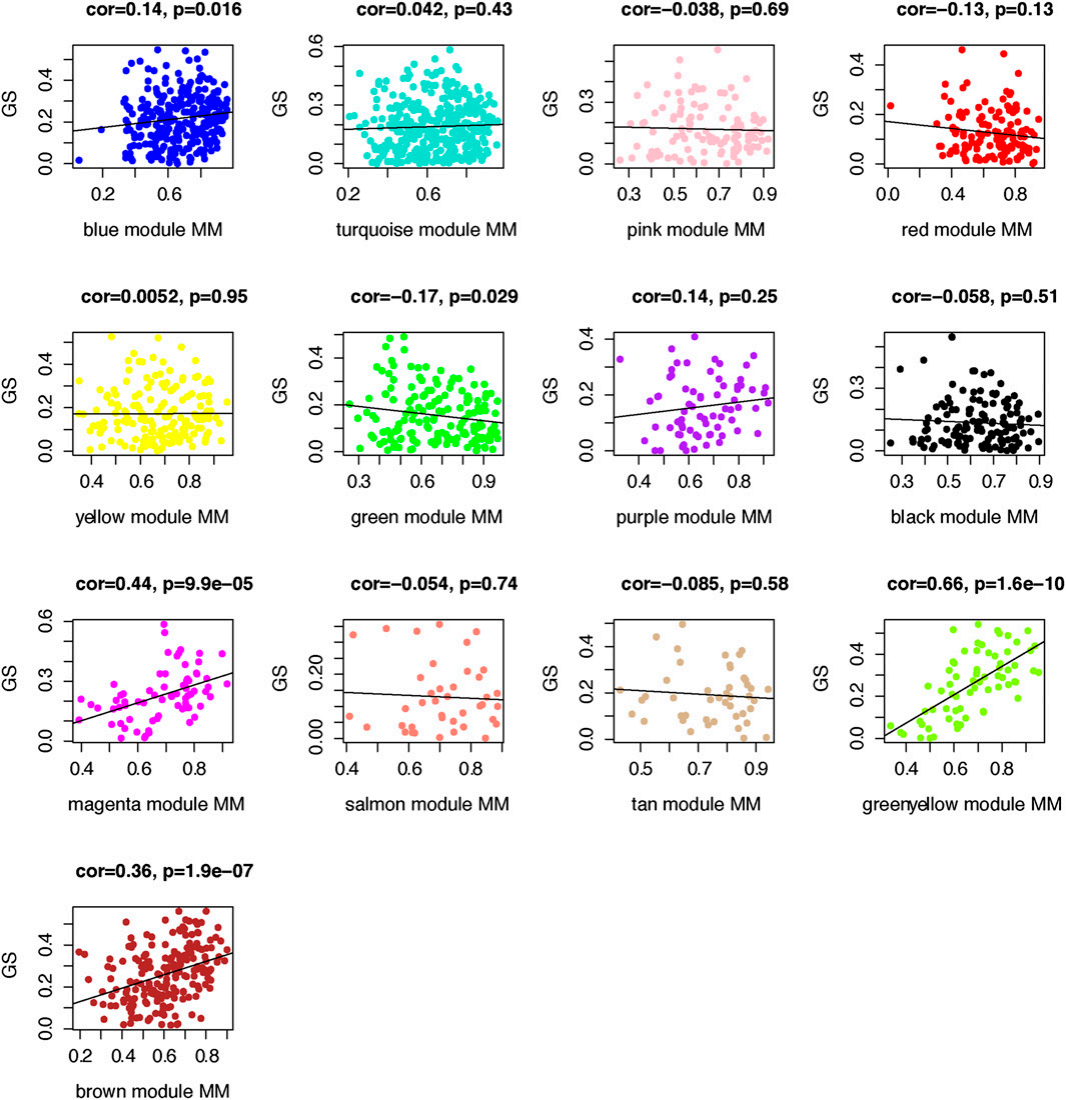
Correlation between MM and GS for each of the 13 distinct GWAS modules. MM (defined as the correlation between each gene’s expression and its module eigengene) for each module is plotted against GS (defined as each gene’s correlation with BMD status). MM in the blue, magenta, greenyellow and brown modules is significantly (*P* < 0.003) correlated with GS.

We focused on the association between the network concept MM and GS, a measure of biological relevance. GS was defined as the absolute value of the correlation between a gene’s expression and BMD status. Of the 13 network modules, significant (*P* < 0.003 after adjusting for number of modules) positive correlations were observed between MM and GS in the magenta (r = 0.44, *P* = 9.9 × 10^−5^), greenyellow (r = 0.66, *P* = 1.6 × 10^−10^), and brown (r = 0.36, *P* = 1.9 × 10^−7^) modules (Figure 5).

On the basis of the correlations between MM and GS, we hypothesized that hub genes from these three modules were the most biologically relevant and thus, the most likely to represent true positive associations with BMD. If true this suggests that selecting genes based on MM may result in greater replication success rates in subsequent studies compared with selecting genes using the traditional metric, GWAS *P*-value. To test this we performed an *in silico* replication study using data from a second BMD GWAS [FOS (Kiel *et al.* 2007)]. Of the 1918 total network genes, 1264 were annotated in FOS using ProxyGeneLD. Genes were considered successfully replicated if their gene-wide associations were less than the significance thresholds defined below with any one of three BMD traits (femoral neck, lumbar spine, and trochanter BMD). From the 1264 network genes annotated in both studies, we compared the FOS replication rates for three groups of genes: (1) hub genes (based on k.in) from the magenta, greenyellow, and brown modules; (2) network genes ranked on GS; and (3) network genes ranked on *P*-value in the deCODE GWAS. The replication rates were compared for the top 20%, 10%, and 5% of genes within each group at three different significant levels, *P* ≤ 0.05, *P* ≤ 0.01, and *P* ≤ 0.001. As shown in Table 5, selecting genes on K.in resulted in greater replication rates in all comparisons. The difference in replication rate between K.in and GWAS *P*-value increased as the definition of a hub gene became more stringent. For example, when comparing the top 5% of hubs *vs.* the top 5% of genes based on *P*-value, the difference in replication rate was twofold higher for hubs. Although validation studies will be needed, these data suggest that k.in may be a better metric than GWAS *P*-value to use to select genes for subsequent replication studies.

**Table 5:** Replication rates of network genes selected using intramodular connectivity (k.in), gene significance (GS), or *P*-value

	Top 20%^a^			Top 10%			Top 5%		
	0.05	0.01	0.001	0.05	0.01	0.001	0.05	0.01	0.001
K.in	35.0%	15.0%	2.5%	57.9%	26.3%	5.3%	60.0%	10.0%	10.0%
GS	35.0%	2.5%	0.0%	21.4%	5.3%	0.0%	20.0%	0.0%	0.0%
*P*-value	32.0%	14.6%	0.0%	34.1%	17.5%	0.0%	30.1%	9.5%	0.0%

a Genes selected for replication were in the top 20%, 10%, and 5% based on K.in or GS in the magenta, greenyellow, and brown modules or *P*-value using all network genes.

## Discussion

In this study, we have applied network theory to a list of genes with evidence of association with BMD using disease-relevant microarray gene expression data in subjects with known BMD status. We demonstrate that network analysis can group genes into modules that are enriched for specific biological processes. In some cases the enrichments were unique to modules and were more detailed and specific than those identified in the entire gene set. We also show that module topology can be used to identify groups of interconnected genes strongly associated with a clinical trait. Not only can this approach be used to reveal hidden enrichments, but it can also identify potentially important coexpression relationships for genes that exceed genome-wide significant thresholds or that have been previously associated with the disease. We also demonstrate that for three of the modules there was a significant correlation between MM and GS. We go on to provide evidence suggesting that hub genes replicate at a higher rate relative to genes selected using GWAS *P*-value or GS. This study provides a framework for combing network analysis and gene expression data to extract additional biological information from GWAS data.

One of the limitations of GWAS is that it does not provide functional information for associated genes. Our systems-level approach does so by grouping genes using expression data from a cell type or tissue that is relevant to the disease in subjects with clinical data. Our discovery of the turquoise submodule of eight genes negatively correlated with BMD is a good example. Importantly, the interconnections between genes in this group could only have been identified by studying their relationships in a disease context. This information combined with the knowledge that they are expressed in mouse osteoclasts can be used to guide *in vitro* and *in vivo* experiments to validate their role in bone.

The major bottleneck in any analysis using GWAS data are generating gene lists. Because of the nature of GWAS data, many SNPs with nominally significant *P*-values will be false-positives. This coupled with the difficulties in converting SNP-based to gene-based *P*-values leads to gene lists that contain a considerable level of noise. What is clear from this study and others (Hong *et al.* 2009) is that potential biases have to be taken into consideration. In addition, our data suggest that functional grouping using coexpression similarities is an excellent approach to separate noise from real biological signal. We have proven this by identifying that the inherent network concept MM is correlated with GS in three of the 13 modules.

The main purpose of any analysis designed to mine GWAS data are the generation of testable hypotheses. We believe a systems-level approach offers many advantages over other strategies for this purpose. For example, we demonstrate that parsing GWAS gene lists into functional groups identified a key role for oxidative phosphorylation, which can now be experimentally validated. Additionally, we identified novel genes based on their connection to known bone genes, membership in an enriched pathway or connectivity in one of the modules in which MM was correlated with BMD. Such genes can be tested to validate their associations and to investigate their biological role in functional genomics and replication studies.

Oxidative stress is known to be increased in age-related diseases such as osteoporosis. It is also known that oxphos plays a direct and key role in bone metabolism (Bratic and Trifunovic 2010; Kousteni 2011). In bone modeling and remodeling, osteoclasts resorb mineral by acidifying the bone matrix (Blair 1998). This process requires significant energetic resources, which are primarily generated through the oxidative phosphorylation of glucose (Williams *et al.* 1997). Recently, it has been demonstrated that increased oxidative phosphorylation occurs in osteoclast precursors as they differentiate into mature osteoclasts (Kim *et al.* 2007). Importantly, our data suggest that genetic variation in multiple oxphos genes influence bone mass. Moreover, the expression of these genes in monocytes is inversely correlated with bone mass, suggesting that increased oxphos in monocytes/osteoclasts results in decreased bone mass.

Our analysis focused on osteoporosis; however, it is likely applicable to any disease with GWAS data and the appropriate gene expression profiles. GWASs have been performed for a myriad of disease. As an example, our search of the Gene Expression Omnibus database at NCBI using the term “cancer” resulted in 344 datasets, suggesting that for many diseases relevant gene expression data that can be used for network analysis is already available.

In conclusion, this study provides proof-of-principle that a systems-level analysis of GWAS data is capable of adding significant value to existing datasets and future studies. This analysis provides a straightforward approach to identify pathways, individual genes, gene modules and network concepts that play an important role in disease.

## Supplementary Material

Supporting Information
