# Supplementary material for: Systems-Level Analysis of Genome-Wide Association Data
Source: G3 (Bethesda). 2013 Jan 1;3(1):119–29. doi: 10.1534/g3.112.004788 (PMC3538337; doi:10.1534/g3.112.004788)
Supplement: Supporting Information [file supp_3_1_119__index.html]

Supporting Information 

# Systems-Level Analysis of Genome-Wide Association Data

## Supporting Information for Farber, 2013

**Files in this Data Supplement:**

- File S1 - Significant (FDR<5%) individual Gene Ontology categories in the NSGG (.xls, 148 KB)
- File S2 - Full list of significant (ES≥3.0) functional clusters in the NSGG (.xls, 169 KB)
- File S3 - Network metrics for all 1918 GWAS network genes (.xls, 545 KB)
- File S4 - List of functional clusters for each GWAS network module (.xls, 1.5 MB)
- File S5 - List of functional clusters for the TNF sub-network (.xls, 83 KB)
